# Disuse Osteoporosis: Clinical and Mechanistic Insights

**DOI:** 10.1007/s00223-021-00836-1

**Published:** 2021-03-18

**Authors:** Tim Rolvien, Michael Amling

**Affiliations:** 1 Division of Orthopaedics, Department of Trauma and Orthopaedic Surgery, University Medical Center Hamburg-Eppendorf, Martinistrasse 52, 20246 Hamburg, Germany; 2 Department of Osteology and Biomechanics, University Medical Center Hamburg-Eppendorf, Lottestrasse 59, 22529 Hamburg, Germany

**Keywords:** Immobilization, Unloading, Bone loss, Microstructure, Osteocyte

## Abstract

Disuse osteoporosis describes a state of bone loss due to local skeletal unloading or systemic immobilization. This review will discuss advances in the field that have shed light on clinical observations, mechanistic insights and options for the treatment of disuse osteoporosis. Clinical settings of disuse osteoporosis include spinal cord injury, other neurological and neuromuscular disorders, immobilization after fractures and bed rest (real or modeled). Furthermore, spaceflight-induced bone loss represents a well-known adaptive process to microgravity. Clinical studies have outlined that immobilization leads to immediate bone loss in both the trabecular and cortical compartments accompanied by relatively increased bone resorption and decreased bone formation. The fact that the low bone formation state has been linked to high levels of the osteocyte-secreted protein sclerostin is one of the many findings that has brought matrix-embedded, mechanosensitive osteocytes into focus in the search for mechanistic principles. Previous basic research has primarily involved rodent models based on tail suspension, spaceflight and other immobilization methods, which have underlined the importance of osteocytes in the pathogenesis of disuse osteoporosis. Furthermore, molecular-based in vitro and in vivo approaches have revealed that osteocytes sense mechanical loading through mechanosensors that translate extracellular mechanical signals to intracellular biochemical signals and regulate gene expression. Osteocytic mechanosensors include the osteocyte cytoskeleton and dendritic processes within the lacuno-canalicular system (LCS), ion channels (e.g., Piezo1), extracellular matrix, primary cilia, focal adhesions (integrin-based) and hemichannels and gap junctions (connexin-based). Overall, disuse represents one of the major factors contributing to immediate bone loss and osteoporosis, and alterations in osteocytic pathways appear crucial to the bone loss associated with unloading.

## Introduction

Skeletal integrity is maintained by the process of bone remodeling, i.e., the balanced removal of old bone matrix by bone-resorbing osteoclasts and the deposition of new bone tissue by bone-forming osteoblasts [1]. Since the early work of Julius Wolff in 1892 [2], it has been well established that the skeleton represents a dynamic tissue undergoing adaptive changes in response to loading to better withstand these loads (“Wolff’s law”). There are several clinical examples of the anabolic response of bone to loading, one of them being the observation that the bone mass is markedly higher in the dominant arm than in the nondominant arm in tennis players [3]. In recent years, a number of essential molecular load-sensation pathways to build the optimal amount of bone required for adequate stability have been uncovered. Osteocytes, which represent terminally differentiated osteoblasts embedded into the bone matrix, have gained research attention due to their ability to sense loading and translate mechanical signals into biochemical signals to orchestrate bone remodeling [4]. The discovery of osteocyte-secreted proteins such as sclerostin has already led to new osteoporosis treatments [5, 6], and more recently, other molecular osteocytic and nonosteocytic proteins and nanostructures involved in the process of mechanosensation, such as the calcium channel Piezo1, were revealed [7–9].

Clinically, disuse represents one of the major risk factors for osteoporosis, a widespread, multifactorial disorder characterized by low bone mass, impaired bone quality and increased risk of fragility fractures [10]. It has been shown that disuse conditions are characterized by an unfavorable combination of high bone resorption and low bone formation [11], leading to immediate bone loss and ultimately to osteoporosis with increased fracture risk [12]. In contrast, skeletal loading leads to an anabolic bone response in terms of increased bone formation [13]. Physical activity represents an effective option to increase bone mineral density (BMD) [14], which is the reason that adequate exercise constitutes the most natural basic treatment for patients with osteoporosis.

The term “disuse osteoporosis” comprises various clinical situations of mechanical unloading or pathological immobilization associated with bone loss, including spinal cord injury [15], neuromuscular diseases [16], bed rest [17], spaceflight [18] and others. Relative unloading has also been discussed as an important factor contributing to the relevant bone loss observed in obese patients after bariatric surgery [19]. Bone loss during disuse or unloading occurs due to a lack of movement and muscular insertions, but it is assumed that lower mechanical stimulation in connection with non-weight-bearing, and especially microgravity, has additional negative effects on skeletal integrity. This is also the reason why bone microstructure studies often compare weight-bearing bones (such as the tibia) with non-weight-bearing bones (such as the radius). The interaction of muscle and bone (“crosstalk”) has opened up a whole new field of science, including both clinical studies on the bone status in sarcopenia in the elderly [20] and, at the basic research level, studies on the interaction of muscle and bone and the role of osteocytes in this relationship [21] (not discussed in detail in this review). This review will focus on recent clinical and molecular advances in understanding the specific effects of unloading on the skeleton. In particular, microstructural changes, mechanistic concepts and options for the treatment of disuse osteoporosis will be discussed.

## Bone Loss under Disuse Conditions

Disuse-associated bone loss has been outlined in many studies, which documented the magnitude of bone loss in a variety of disuse conditions early on. Dual-energy X-ray absorptiometry (DXA), regularly performed for the proximal femur and lumbar spine, represents the most established method to quantify the areal BMD and to determine the diagnosis of osteoporosis or low bone mass based on T- and Z-scores, respectively. DXA studies have been widely performed in disuse conditions, with both cross-sectional and longitudinal study designs.

### Spinal Cord Injury and Neurological/Neuromuscular Disorders

In patients with spinal cord injury, early studies have demonstrated that bone loss begins shortly after injury, with up to 2–4% bone loss per month in the sublesional skeletal sites during the first year [22]. A peak of increased bone resorption was reported at approximately three months after injury [23, 24]. While a steady bone metabolic state at approximately 60–70% of the original bone mass has been observed after 1.5–2 years [25], other studies have documented the persistence of increased bone resorption markers after similar follow-up periods [24]. While other neurological disorders, such as multiple sclerosis [26] and a variety of pediatric neuromuscular diseases [27, 28], are also associated with disuse-induced bone loss, it is likely that unloading is not the only factor causing skeletal deterioration and that other factors (neuronal, immunological, or vascular) also contribute to the observed bone loss. Nonetheless, the fact that the skeletal sites not affected by disuse (e.g., the upper extremities in spinal cord injury) are not affected by bone loss [24, 29] confirms the key role of the mechanical environment, although it is important to note that non-weight-bearing bones can also generally be affected by disuse-induced bone loss, such as in tetraplegia [29], or in the affected limbs in patients after stroke [30].

### Bed Rest

**Fig. 1:**
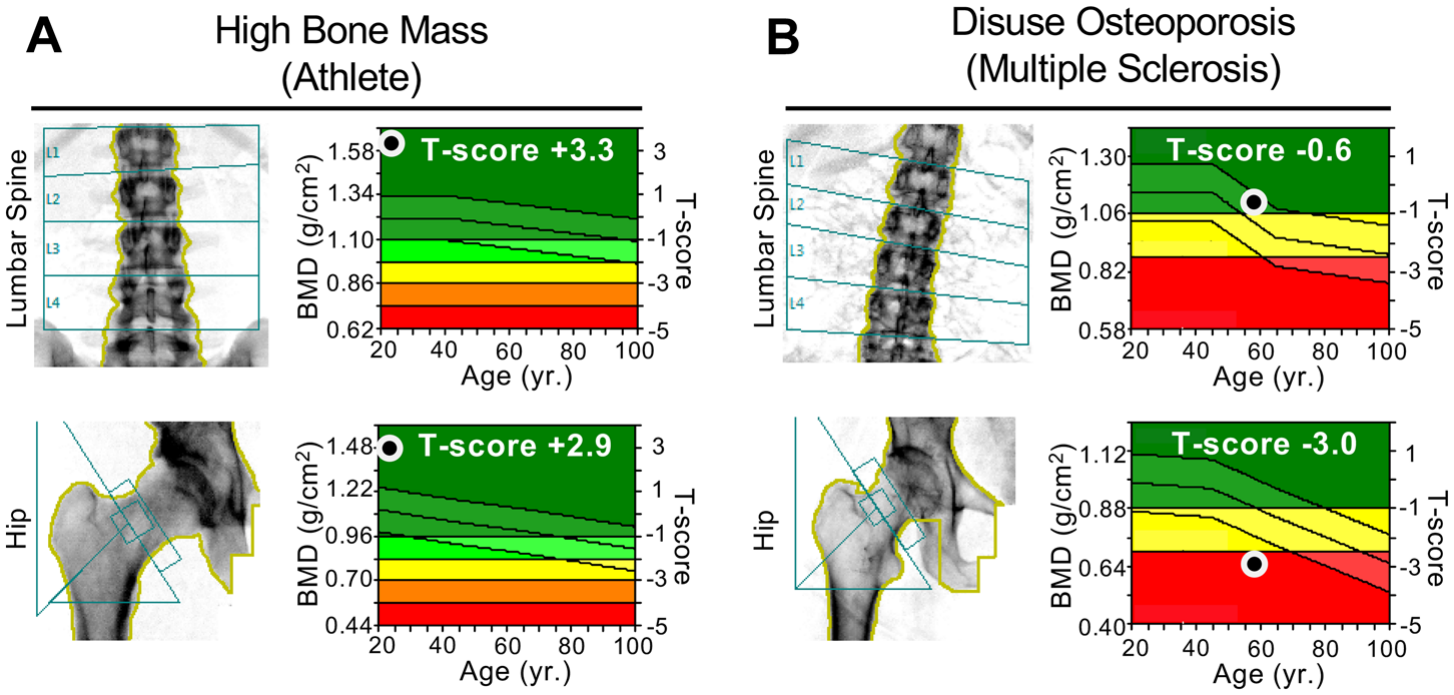
Clinical examples of two patients with high vs. low BMD in response to intense physical exercise vs. unloading. **a** DXA scans of a professional athlete (soccer player) with high BMD values in both the lumbar spine and hip. **b** DXA scans of an immobilized patient with multiple sclerosis showing low BMD values, which were more severe in the hip than the lumbar spine, meeting the World Health Organization’s (WHO) definition of osteoporosis in the hip (i.e., T-score ≤ − 2.5)

To further address the magnitude and mechanisms of disuse-induced bone loss, a number of bed rest studies have been performed, with different approaches. Most studies also aimed to assess the recovery of bone mass in response to reambulation (reloading). In early studies, a similar amount of bone loss in the lumbar spine and hip of approximately 1% per month was detected in healthy males who had been followed up for 17 weeks [31]. Other bed rest studies in premenopausal women also found that the bone loss in the proximal femur was approximately 1% per month, but the bone loss in the lumbar spine was less severe [32]. It is interesting to note that weight-bearing, but not non-weight-bearing skeletal sites such as the forearm, showed significant bone loss during bed rest [33], which underlines the additional influence of weight-bearing. Our clinical experience in treating patients with skeletal disorders and osteoporosis supports the notion that disuse affects weight-bearing bones more severely than non-weight-bearing bones or the axial skeleton, such as the vertebral column (Fig. 1). Finally, bone loss has also been documented by DXA in elderly patients with disuse conditions, including patients who were bedridden or in a vegetative state, although these studies did not comment on the exact rate of bone loss per unit time [34, 35].

### Spaceflight

In addition to bed rest, the loss of BMD has also been well documented in spaceflight studies. Here, the rate of bone loss ranged from 1.07–1.92% per month in the proximal femur and 0.51–0.68% per month in the spine [36], or in other studies, 1.4–1.5% per month in the proximal femur and 0.9% per month in the spine [37]. While microgravity-induced bone loss is positively associated with the duration of spaceflight, the lack of complete recovery or even persistence of bone loss after return to Earth is both unfavorable and not fully understood [18, 38].

### Local Disuse Osteoporosis

**Fig. 2:**
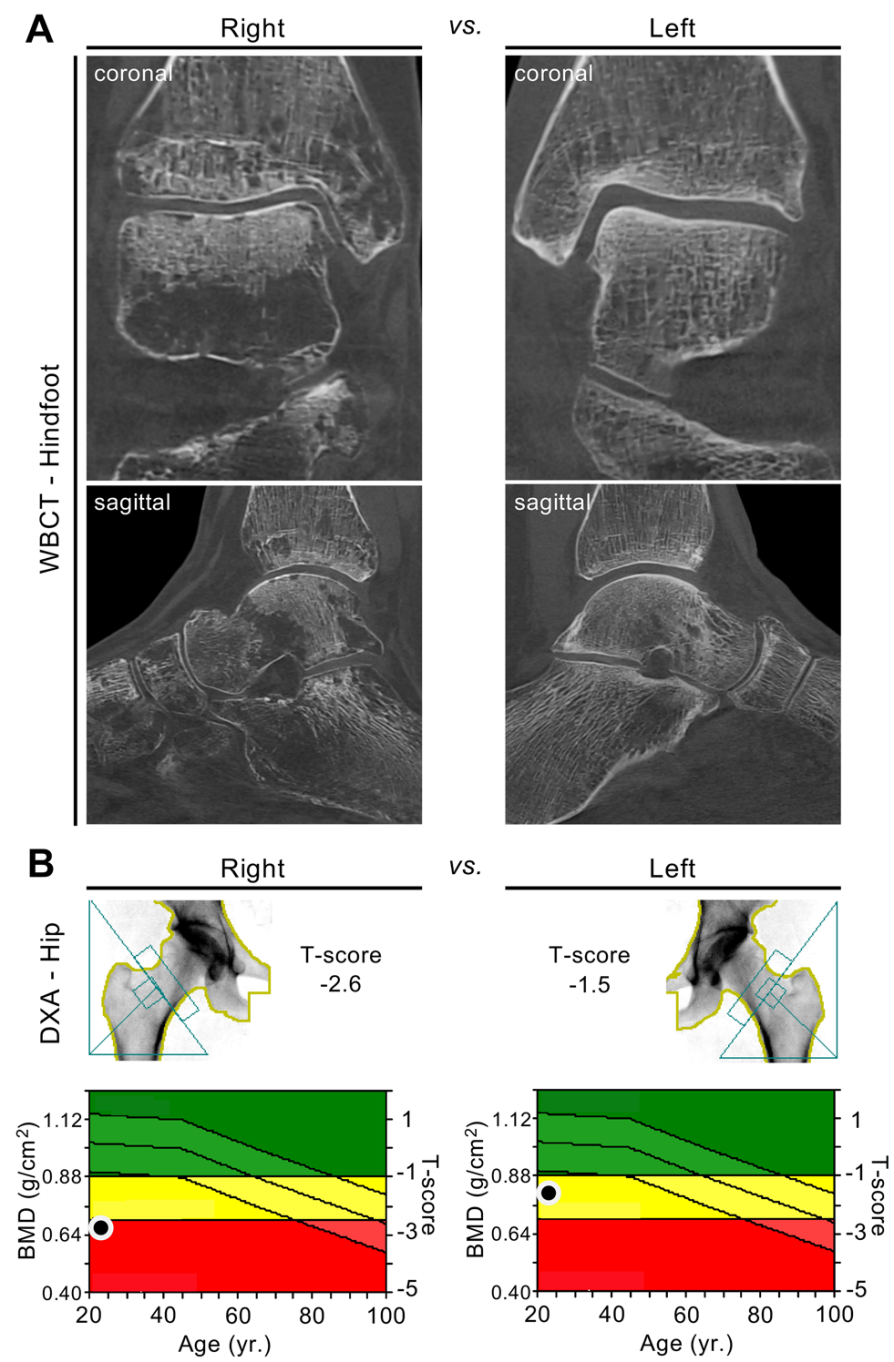
Local deterioration of bone mass after fracture and subsequent unilateral disuse of the right limb. **a** Cone-beam computed tomography (CBCT) images of the right foot compared to the left foot (coronal and sagittal reconstruction) in a 22-year-old female patient obtained eight months after suffering from an ankle fracture with subsequent unilateral unloading of the right limb. Focal osteolytic changes (often referred to as “local disuse osteopenia/osteoporosis”) are visible. **b** DXA scans demonstrating the differences in BMD between the right and left proximal femur (T-score − 2.6 vs. − 1.5). Bilateral HR-pQCT scans of the distal tibiae were also performed and showed cortical and trabecular bone loss syndrome on the unloaded side compared to the contralateral (loaded) side (cortical thickness − 28%, bone volume per tissue volume − 7%)

Disuse osteoporosis includes not only systemic bone loss but also various conditions of local bone loss. In a previous study, magnetic resonance imaging (MRI) was performed in local disuse osteoporosis, where high signal intensity in the respective unloaded limbs was detected, indicating immediate changes following unloading [39]. In a cohort of patients after stroke, the affected lower extremities showed lower mineral and geometric characteristics than the unaffected upper extremities [40]. Rapid unilateral bone loss has been observed in patients after surgery [41] and after experimental unilateral limb suspension [42]. The bone loss observed after periods of reduced loading represents a strong risk factor for subsequent fractures [43]. These unilateral changes can be underlined by clinical examples, such as bone loss that is readily visible on CT images and measurable by DXA after fracture with subsequent immobilization (Fig. 2**).**

### Laboratory Changes in Bone Turnover and Calcium Metabolism

In previous clinical studies focusing on laboratory changes in bone turnover, disuse or unloading was associated with increased bone turnover (primarily bone resorption) across different disuse conditions such as spinal cord injury [23, 24], vegetative state [35] or spaceflight [38]. While some studies, including bed rest [44] and spaceflight [38] studies, failed to detect any changes in bone formation, other bed rest studies found low bone formation marker levels accompanied by increased sclerostin levels [11, 45]. Patients with nonambulatory (i.e., immobilized) cerebral palsy showed higher sclerostin levels than patients with ambulatory cerebral palsy [46]. In contrast to these previous findings, one study found that sclerostin levels were lower in subjects with spinal cord injury who were immobilized in a wheelchair than in those who walked regularly [47]. Sclerostin represents a protein that is almost specifically produced by mechanosensitive osteocytes [5], which highlights the potential role of osteocytes in the pathogenesis of disuse osteoporosis [11, 45, 48]. Conversely, sclerostin levels were lower and bone formation was higher with higher physical activity [49]. Sclerostin inhibition prevented spinal cord injury-induced cancellous bone loss in a rodent spinal cord injury model [50]. However, the effects of sclerostin inhibition on bone quality in human patients suffering from disuse osteoporosis conditions have not yet been examined in detail. Since the sclerostin antibody romosozumab is now an established and approved treatment for severe postmenopausal osteoporosis [6], it will be of interest whether this drug is particularly effective in disuse osteoporosis or, more generally, how effective the drug is under different loading conditions.

Along with these previous findings on catabolic bone turnover dissociation following disuse, it is clear that the organism’s calcium metabolism is also impacted. Urinary calcium excretion was increased in patients during bed rest [45] and in individuals during spaceflight [51]. Furthermore, vitamin D deficiency was observed more frequently in immobilized patients in a vegetative state than in controls [35]. Interestingly, the high calcium loss observed during bed rest could not be counteracted by higher calcium intake [48]. These collective findings point to a specific pattern of high bone resorption and low bone formation during disuse, which is accompanied by calcium loss and, in most conditions, also vitamin D deficiency.

### Fracture Healing

The impact of mechanical load on the process of fracture healing represents a complex issue that must be considered separately from the aspects of disuse-induced alterations in bone remodeling outlined in this review. Nonetheless, it is known that the compromised mechanical environment associated with immobilization and load-bearing restrictions in the course of operative or conservative fracture treatment may be followed by a vicious cycle of disuse-induced fracture healing problems. While direct bone healing is usually achieved by high stability and low interfragmentary movement (e.g., by compression plates), indirect bone healing via callus formation is induced by a low degree of stability and considerable interfragmentary movement (e.g., by internal or external splinting) [52]. Therefore, the most adequate balance between fracture stability and mobilization or load-bearing is directly influenced by the anatomical fracture localization and/or the surgical concept [53]. During indirect fracture healing, controlled loading such as cyclic compression of fractures led to enhanced callus formation [54], but optimal loading protocols (e.g., timing, strain) are still debated. In conditions of reduced mechanical loading, osteoanabolic teriparatide (PTH 1–34) treatment could be a promising option to improve fracture healing, as evidenced by mouse studies [55]. PTH administration together with loading across the fracture site was associated with further accelerated fracture healing, indicating a synergistic effect [56].

## Microstructural Findings

To obtain more detailed data on the bone loss associated with disuse beyond DXA-based observations, volumetric and microstructural approaches, primarily peripheral quantitative computed tomography (pQCT) and, more recently, high-resolution peripheral quantitative computed tomography (HR-pQCT), have been introduced. The bone microstructural changes following immobilization have also been discussed in review articles [57], including those specifically discussing bone loss patterns in patients with spinal cord injury [58] and other neuromuscular disorders [59].

Previous pQCT studies with cross-sectional study designs have demonstrated negative associations between the duration of disuse and bone microstructural parameters in patients with spinal cord injury, where steady states were reached after 3–8 years and the total amount of bone loss ranged from 25–35% (diaphyses) to 50–60% (epiphyses) [60]. Only a few studies with longitudinal approaches, i.e., repeated measurements of the same parameters and in the same patients at standardized time intervals after disuse onset, are available. A selection of relevant longitudinal pQCT and HR-pQCT studies is presented in Table 1. Simulated (i.e., bed rest) or real microgravity studies are typically performed in young and healthy individuals. These conditions have been shown to lead to a pattern of both trabecular and cortical bone loss [37, 57]. During bed rest, it was reported that the relative bone loss in the tibia was larger in the cortical than the trabecular compartment [61]. Furthermore, relevant site specificity was demonstrated, with all tibial regions (epiphyseal to diaphyseal) depicting cortical loss, while considerable trabecular loss could only be detected in the proximal tibia epiphysis. However, a similar team of authors also observed greater trabecular loss in the distal tibial epiphysis in another bed rest study [62]. Greater trabecular than cortical loss was also detected after acute spinal cord injury [29]. More recently, HR-pQCT bed rest studies have confirmed that bone loss in both male and female individuals occurs primarily in weight-bearing bones such as the tibia [63, 64]. These studies demonstrated combined cortical and trabecular bone loss during bed rest.

**Table 1:** Relevant studies on microstructural findings (pQCT, HR-pQCT) under real or modeled disuse osteoporosis conditions

Study	Model/Sex	Method	Skeletal site	Disuse/Recovery	% Loss	% at Recovery
Rittweger et al. [61]	Bed rest/Male	pQCT	Tibia, Patella, Femur	35 days	BMC − 0.7 to − 3.2% (mostly cortical, epiphyseal)	n/a
Rittweger et al. [67]	Bed rest/Male	pQCT	Tibia, Radius	56 days/360 days	Tib.BMC − 3.6% (dist. epiphysis)	Tib.BMC − 1.4% (dist. epiphysis)
Beller et al. [32]	Bed rest/Female	pQCT	Tibia, Radius	43 days/360 days	Tib.vBMD − 1.9% Rad.vBMD − 0.2%	Tib.vBMD − 1.8% Rad.vBMD − 1.4%
Belavy et al. [63]	Bed rest/Male	HR-pQCT	Tibia, Radius	59 days/720 days	Tib.Ct.Th − 2.2% Tib.Tb.N − 4.2% Rad.Ct.Th + 1.0% Rad.Tb.N + 1.3%	Tib.Ct.Th − 0.4% Tib.Tb.N − 3.7% Rad.Ct.Th + 1.2% Rad.Tb.N + 1.6%
Armbrecht et al. [64]	Bed rest/Female	HR-pQCT	Tibia, Radius	43 days/360 days	Tib.Ct.Th − 1.5% Tib.Tb.N − 1.4% Rad.Ct.Th − 0.1% Rad.Tb.N − 2.3%	Tib.Ct.Th − 0.6% Tib.Tb.N − 3.1% Rad.Ct.Th − 0.4% Rad.Tb.N − 3.0%
Vico et al. [38]	Spaceflight/Male	HR-pQCT	Tibia, Radius	4–6 mo./12 mo.	Tib.Ct.Th − 3.5% Tib.Tb.N − 4.6% Rad.Ct.Th − 1.1% Rad.Tb.N + 4.9%	Tib.Ct.Th ± 0% Tib.Tb.N − 4.6% Rad.Ct.Th − 4.5% Rad.Tb.N + 1.5%
Coupaud et al. [29]	Spinal cord injury/Male + Female	pQCT	Tibia, Femur, Radius	12 mo.	Tib.Tb.BMD − 17.3 to – 22.2% Tib.Ct.BMD − 2.5 to – 2.6% Rad.Tt.BMD − 1.4%	n/a
Rittweger et al. [42]	Unilateral limb suspension/Male	pQCT	Tibia	24 days/90 days	Tib.BMC − 0.21 to – 0.6%	BMC − 0.52 to – 0.64%
Kazakia et al. [41]	Surgery/Male + Female	HR-pQCT	Tibia	6 weeks/13 weeks	Ct.Po + 16.1% Tb.N + 5.6% Tb.Th − 5.4%	Ct.Po + 16.2% Tb.N recovered* Tb.Th recovered*

A selection of relevant microstructural human studies with longitudinal data from 2000–2020 is presented. The table includes the magnitude of both bone loss and recovery (the displayed data are for the respective control groups, while the therapeutic effects of some studies are not displayed). The results are sorted by disease/model and year

*Tib*. tibial, *Rad*. radial, *BMC* bone mineral content, *vBMD* volumetric bone mineral density, *Tt.BMD* total bone mineral density, *Ct.BMD* cortical bone mineral density, *Tb.BMD* trabecular bone mineral density, *Ct.Th* cortical thickness, *Tb.N* trabecular number, *Tb.Th* trabecular thickness, *Ct.Po* cortical porosity, *n/a* not available/not assessed

*Not reported as numeric values

During spaceflight, cortical and trabecular bone loss was observed in the tibia [65]. Notably, trabecular bone showed both more severe and earlier bone loss than cortical bone, although a high degree of variability among the subjects was noted. There have also been QCT studies with measurements of the lumbar spine and hip, in which trabecular and cortical bone loss has been documented [37]. A recent HR-pQCT study in subjects before and after spaceflight showed that the loss of cortical bone was not only characterized by cortical thinning but also primarily by an increase in cortical porosity of 15% at the distal tibia [38]. Interestingly, a similar increase in cortical porosity (+16.1%) was also observed in the affected limbs of patients after 6 weeks of non-weight-bearing following surgery [41]. In summary, both trabecular and cortical compartments are affected by disuse, and while site and compartment differences exist among the disuse conditions, the most pronounced bone loss seems to occur in the epiphyses, with cortical porosity being the microstructural parameter that has shown the highest increases.

## Recovery and Therapeutic Considerations

The recovery of bone mass in response to physical exercise programs, pharmacotherapy or both has been studied across different disuse and unloading models. Indeed, the most natural treatment for disuse osteoporosis is physical exercise or remobilization (loading) of the affected bones, although some of the disuse conditions per se do not allow remobilization. A recent systematic review found that the favorable effect of exercise on BMD in postmenopausal women is largely independent of the type of exercise [66]. Experiments on recovery from disuse conditions (mostly bed rest) often compare a study group with different exercise programs during or after immobilization with a control group (i.e., the study group without any additional exercise program). It is interesting to note that bed rest-induced bone loss continues in the first days/weeks after reambulation [61, 67]. In different control groups, the bone mass did not fully recover within the same time that the individuals were immobilized and, in most studies, even after long-term follow-up [32, 67] (Table 1). For instance, a remaining significant reduction in the tibial epiphyseal bone mineral content (BMC) was noted at approximately one year after reambulation [67]. While HR-pQCT bed rest studies have indicated a recovery of most microstructural parameters (including the cortical thickness or trabecular bone volume fraction) after mid-term follow-up, the trabecular number in the distal tibia remained significantly reduced after two years of follow-up in both male and female subjects [63, 64]. In individuals returning from space, the tibial cortical density recovered, while the cortical porosity or trabecular bone did not fully recover during the year after landing [38]. Increased cortical porosity after 6 weeks of non-weight-bearing did also not recover after 13 weeks of full weight-bearing [41].

Regarding the optimal exercise program, various studies with different protocols have been conducted, from which diverging findings regarding their success could be derived. In individuals with spinal cord injury, an overall positive and site-specific effect of functional electrical stimulation-induced cycling exercise on bone mass has been observed [68–70]. These studies also suggested a dose-dependent effect, with the best results after high-volume training [68]. To counteract bone loss during bed rest, the addition of whole-body vibration to high-load resistive exercise has been found to be effective in male subjects [67]. HR-pQCT studies have further confirmed these findings, with the combination of vibration and resistive exercise showing overall better preservation of cortical and trabecular parameters than resistive exercise alone [71]. In women, a previous pQCT study found that a combination of resistance and aerobic training did not provide any benefit compared to the control protocol [32]. In another study, this was also confirmed by HR-pQCT, where combined resistive and endurance exercise countermeasures were ineffective in preventing skeletal deterioration during disuse in women [64]. Such findings suggest a sex-specific effect in the skeletal response to loading, and the difference in the loading response may be attributed to sex hormones (estrogens and androgens) and their receptors [72]; however, the exact mechanisms are complex and remain partly unexplored. Of note, previous studies in non-immobilized patients examining the treatment effects of whole-body vibration on BMD have shown conflicting results with both positive and no effects [73, 74].

The fact that increased bone resorption plays a primary role in the development of disuse osteoporosis is supported by the findings of several previous therapeutic studies, in which antiresorptive treatment with bisphosphonates or the receptor activator of NF-κB ligand (RANKL) antibody denosumab attenuated bone loss [44, 75]. For example, intravenous bisphosphonates were partly effective in preserving bone mass in male subjects during bed rest [76]. Furthermore, denosumab treatment led to preserved bone mass in the knee region in patients with subacute spinal cord injury [75]. Compatible with the observation that chronic unloading also leads to low bone formation, the osteoanabolic agent teriparatide led to a solid increase in BMD in individuals with chronic spinal cord injury [77]. Interestingly, this increase was not augmented by additional vibration stimulation [77]. A previous study in postmenopausal, non-immobilized, osteoporotic women demonstrated a clinically relevant treatment effect in lumbar spine BMD with twelve months of whole-body vibration exercise and teriparatide compared to teriparatide alone [78].

Taken together, the treatment of disuse osteoporosis by combining bone-specific medications with physical exercise appears to be effective in at least partially counteracting the observed bone loss. In particular, the prompt increase in bone resorption markers after the onset of disuse [24] argues for the early use of anti-resorptive drugs such as denosumab. Individual exercise programs using vibration or electrical stimulation have shown positive, but sometimes inconsistent, results. The combination of bone anabolic drugs with physical exercise protocols appears to be promising but remains largely unexplored in conditions of disuse osteoporosis.

## Basic Research on Skeletal Mechanotransduction

**Fig. 3:**
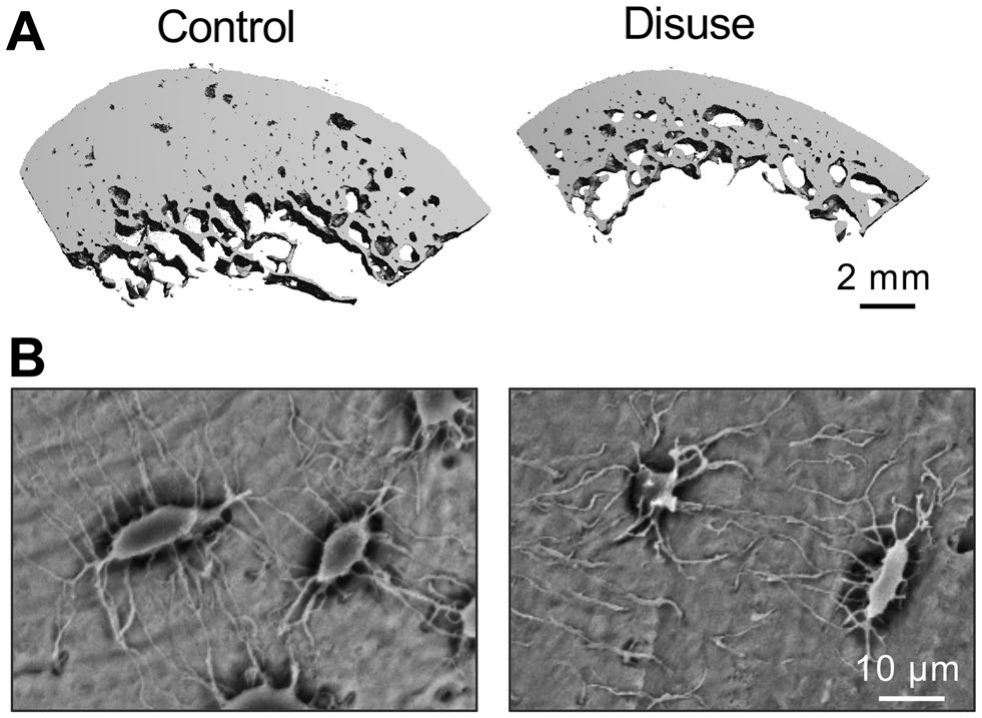
Micromorphological changes in cortical bone after immobilization. **a** µ-CT images demonstrating profound cortical changes, including a decrease in cortical thickness and an increase in cortical porosity, in individuals after long-term bed rest. **b** SEM images of acid-etched plastic-embedded cortical bone sections revealing decreased canalicular connectivity of osteocytes after immobilization, underlining the role of osteocytes in mechanotransduction. Images were obtained from representative samples obtained in the context of a previous study [79]

To decipher the mechanisms of disuse-induced bone loss, loading and unloading conditions have been modeled in a variety of human and animal studies. In one of our previous experimental human cadaveric studies analyzing the micromorphology of cortical bone, we found increased cortical porosity in long-term immobilization compared to controls and postmenopausal osteoporosis [79] (Fig. 3a), which supports previous clinical findings of a primary effect on cortical porosity after disuse [38]. Cortical deterioration was associated with lower osteocyte viability and impaired osteocyte connectivity [79] (Fig. 3b).

Regarding the evaluation of recovery or therapeutic effects in disuse osteoporosis, there have been many experimental studies taking advantage of animal models. One such study reported that different types of exercises (e.g., swimming, jumping, or vibration) applied to rats with immobilization-induced bone loss were equally efficient in preventing bone loss [80], thereby confirming clinical observations. Disuse in rats attenuated the bone anabolic response to teriparatide treatment [81]. In another experimental study in dogs, the cortical bone loss observed in disuse osteoporosis could only partially be counteracted by bisphosphonates, which indicates that the strong osteoclastogenic stimulus of disuse may not fully be antagonized by antiresorptive agents [82]. Together, these results suggest that mechanical loading is of critical importance in optimizing the treatment effects of bone-specific agents. Of note, an emerging model to study bone and its adaption to physical activity is the zebrafish [83]. Modification of the physical exercise intensity in a swim tunnel experiment led to rapid bone formation and increased bone volume and mineralization [84].

There have been a number of studies in rodents focusing on mechanical loading, bone remodeling and especially osteocyte mechanobiology. In mice, spaceflight led to both trabecular and cortical deterioration along with increased numbers of cortical empty osteocyte lacunae [85]. Early studies found evidence of osteocytic changes following immobilization, including increased osteocyte apoptosis [86]. More recently, it was demonstrated that osteocyte apoptosis following weightlessness (i.e., unloading by tail suspension) in mice triggers osteocytic RANKL production along with activation of both cortical and trabecular bone resorption [87]. While osteocyte apoptosis preceded the recruitment of osteoclastic cells and bone loss [88], another study by the same group found that inhibition of osteocyte apoptosis did not prevent unloading-induced bone loss although osteocytic RANKL production was sufficiently inhibited [89]. Tatsumi et al. developed a transgenic mouse model with the ablation of osteocytes through the injection of diphtheria toxin [90]. Importantly, these mice did not develop unloading-induced bone loss, underlining the osteocyte’s function in sensing mechanical loading and in the development of disuse osteoporosis. It is interesting to note that other models, including Botox-induced immobilization, were shown to lead to significant changes in bone mechanical and structural properties without affecting cortical osteocyte lacunar properties in rats [91]. A reason for these diverging findings, and especially the lack of osteocytic changes in the latter work, may be due to the almost nonexistent intracortical remodeling in the rats studied as well as the fact that osteocyte apoptosis was not studied [91]. Overall, it is likely that certain factors influence how osteocytes respond to unloading, for example, cortical vs. trabecular bone compartments, local loading history, and remodeling rates.

**Fig. 4:**
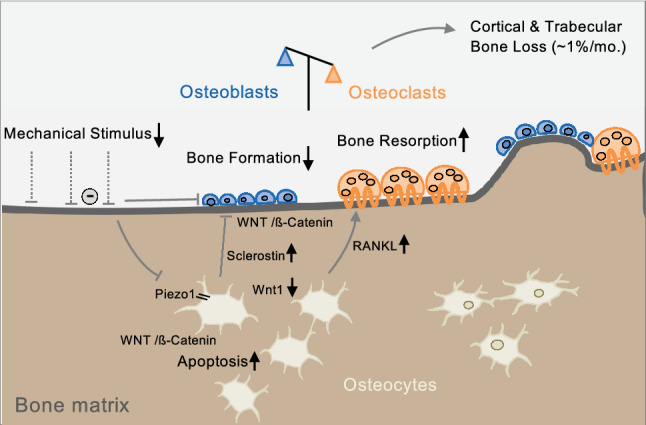
Schematic model of the molecular pathways involved in skeletal unloading. Mechanosensation in osteocytes is mediated by the lacuno-canalicular system (LCS) and via ion channels of the Piezo family (primarily Piezo1), among others. Inactivation of Piezo1 leads to decreased Wnt1 expression. Furthermore, sclerostin secretion by osteocytes is increased in response to unloading, which inhibits Wnt/β-catenin and results in suppressed osteoblast activity. Unloading also leads to increased osteocyte apoptosis (partly via Wnt/β-catenin signaling) and increased RANKL expression, promoting increased bone resorption

In the search for molecular mechanisms involved in the pathogenesis of disuse osteoporosis, several other important signaling pathways and proteins involved in the process of mechanically driven bone remodeling have been discovered (Fig. 4). Indeed, matrix-embedded osteocytes represent the skeletal cell type that most likely plays a major role during mechanotransduction, i.e., the process of converting external mechanical forces into biochemical responses. This is achieved by sensing local mechanical signals and responding to these signals both directly and indirectly [92]. Notably, both mechanically induced bone formation and disuse-induced bone loss and skeletal fragility may be mediated by osteocytes. Osteocytes are embedded in a lacuno-canalicular system (LCS), which becomes evident in acid-etched bone with subsequent scanning electron microscopy. Inside the LCS reside the osteocytes and the dendritic processes that originate from the osteocyte cell body. The osteocyte-secreted protein sclerostin was originally discovered through the identification of loss-of-function mutations in the SOST gene in individuals with the genetic high-bone-mass disorder sclerosteosis (van Buchem disease) [93]. Further research has demonstrated that increased sclerostin expression is primarily caused by mechanical unloading in mice, which leads to low bone formation [94]. While in vitro studies have revealed that disuse promotes osteocyte apoptosis and mechanical stimulation by fluid shear stress promotes osteocyte survival [95], it has further been demonstrated that increased sclerostin expression is specifically caused by unloading in osteocytic cells [96]. Translating these findings into therapeutic concepts, it is most likely that the recently approved sclerostin antibody romosozumab [6, 97] is especially effective in disuse osteoporosis, but the efficacy of romosozumab in association with conditions of disuse osteoporosis has to be investigated in future clinical studies.

The mechanosensitive function of the skeleton was recently evidenced by the identification of mechanically activated ion channels of the Piezo family, specifically Piezo1 in osteoblasts and osteocytes [7, 8]. Interestingly, the deletion of Piezo1 reduced the expression of Wnt1 [7], which is a key regulator of bone formation [98]. By a systematic analysis of mice with inactivated Piezo proteins in different skeletal cell populations, our group confirmed that Piezo1 acts as a mechanosensor in osteocytes and demonstrated that it represents an essential osteogenic differentiation factor during endochondral bone formation [9]. The latter finding supports the assumption that various cell types are involved in mechanotransduction, which is highly relevant not only for bone remodeling but also for developmental and regenerative processes [99]. Together, these collective data suggest that Piezo1 plays a crucial role in mechanosensation and that Piezo channels could be a novel therapeutic target for osteoanabolic treatment in mechanical unloading-induced bone loss.

Another molecular example of musculoskeletal mechanotransduction, more specifically the close interaction between muscle and bone during this process, is the identification of the myokine irisin, a protein that is derived from muscle in response to exercise. Irisin injection increased cortical bone mass in mice, partly through suppression of sclerostin expression [100]. In this way, irisin prevented not only muscular atrophy but also the development of disuse osteoporosis accompanied by decreased osteocyte apoptosis [101]. In human subjects, circulating irisin levels were associated with the risk of osteoporotic fracture [102]. Beyond this associative evidence, there is currently no strong clinical evidence demonstrating that muscle or bone modulate each other’s tissue response to load changes.

Finally, the molecular mechanosensors in osteocytes are the subject of ongoing research, as recently discussed in a detailed review article [92]. Briefly summarized (and as partly outlined above), osteocytic mechanosensors include the osteocyte cytoskeleton and dendritic processes within the lacuno-canalicular system (LCS), as well as ion channels (e.g., Piezo1). However, the extracellular matrix (ECM), primary cilia, integrin-based focal adhesions and connexin-based intercellular junctions have also been identified to contribute to mechanotransduction in osteocytes. During skeletal development and mechanical stimulation, there is interactive communication between osteocytes and the ECM, which is mainly composed of proteoglycans, glycoproteins, and hyaluronic acid. One example highlighting the role of the ECM during the osteocyte mechanotransduction process is perlecan (HSPG2), a proteoglycan that is localized in the pericellular space of osteocytes within the LCS [103]. Importantly, perlecan-deficient mice showed dysfunctional bone formation in response to loading [104]. Primary cilia are solitary, rigid structures that span from the cell body into the extracellular space [105]. Interestingly, primary cilia translate extracellular mechanical stimuli into cellular responses via an ion-channel-independent mechanism [106]. In addition to these important structures responsible for mechanotransduction in osteocytes, integrin-based focal adhesions play an important role in this complex process. Integrins are transmembrane receptors with an extracellular and a cytoplasmic domain composed of an α and β subunit. Both in vitro and in vivo studies have revealed the involvement of the different subunits during mechanotransduction [107]. Finally, cellular communication via hemichannels and gap junctions, which are composed of the protein connexin, has been found to be essential during mechanotransduction. While multiple types of connexins are expressed in bone cells, Cx43 is the major candidate for mechanotransduction in osteocytes. The cell-specific deletion of Cx43 in osteoblasts and osteocytes led to an attenuated response to both loading and unloading [108, 109].

## Concluding Remarks

Although a number of fundamental questions concerning the clinical, molecular and therapeutic aspects of disuse osteoporosis remain unanswered, the combined effort of both clinical and basic research has revealed the microstructural and mechanistic basis of the profound negative effects of disuse on skeletal integrity. Clinical studies with real or modeled unloading conditions, including spinal cord injury, limb suspension, bed rest and microgravity (spaceflight), have demonstrated that early bone loss is characterized by a relative increase in bone resorption compared to decreased bone formation. As a consequence, combined cortical and trabecular microstructural deterioration was noted in most studies, with subtle disease-, sex-, site- and compartment-specific differences that can be accurately imaged using high-resolution CT-based techniques such as HR-pQCT. In a broader sense, disuse osteoporosis also includes clinical conditions of bone loss in response to local skeletal unloading, such as after surgery or around implants. Furthermore, full and timely recovery from bone loss may not be achieved by loading, but bone loss may be counteracted by high-frequency physical exercise programs and bone-specific agents such as bisphosphonates/denosumab and teriparatide. The use of animal models has allowed the cellular and molecular mechanisms of disuse-induced bone loss to be elucidated. Matrix-embedded osteocytes have been identified as the primary mechanoresponsive cell type translating mechanical into biochemical signals to orchestrate mechanically induced bone formation and remodeling, which is in fact a regulatory process with high complexity also involving neighboring bone cells. Osteocyte-specific proteins such as sclerostin and Piezo1 are involved in this process of mechanotransduction, and new therapeutic options have already been introduced; however, their efficacy needs to be clarified in further experiments and clinical trials acknowledging the mechanical environment.
